# Observation of photobleaching in Ge-deficient Ge_16.8_Se_83.2_ chalcogenide thin film with prolonged irradiation

**DOI:** 10.1038/s41598-017-14796-w

**Published:** 2017-11-03

**Authors:** Sen Zhang, Yimin Chen, Rongping Wang, Xiang Shen, Shixun Dai

**Affiliations:** 1 Laboratory of Infrared Materials and Devices, Ningbo University, Ningbo, Zhejiang, 315211 China; 2 Key Laboratory of Photoelectric Detection Materials and Devices of Zhejiang Province, Ningbo, 315211 China; 3 University of Chinese Academy of Sciences, Beijing, 100049 China; 4 Laser Physics Centre, The Australian National University, Acton, Canberra ACT 2605 Australia

## Abstract

We presented the unusual result of photobleaching (PB) in Ge-deficient Ge_16.8_Se_83.2_ thin films with continuous irradiation with 560 nm laser for 12000 s, which is contradicted with the previous reports that the PB only occurs in Ge_x_Se_100-x_ films with x > 30. Observation of the dynamics variations of the photo-induced effects indicated that, photodarkening (PD) appears almost instantaneously upon light irradiation, saturates faster in a shorter time scale, and then photobleaching (PB) becomes dominant. Moreover, both PD and PB process accelerates with increasing irradiation power density. Raman spectra provided the evidence on the change of the photostructure of the samples, e.g. the structural transformation from Ge(Se_1/2_)_4_ edge-sharing (ES) to corner-sharing (CS) tetrahedral and homopolar Ge-Ge and Se-Se bonds to heteropolar Ge-Se bonds.

## Introduction

Photodarkening (PD) and photobleaching (PB) effects, defined as a red or blue shift in an optical absorption edge, respectively^1,2^, are useful for a variety of applications such as information storage, optical switching and so on^3–5^. Two models alternative can be used to explain the origin of the PD and PB effects: one is assumed from photo-induced structural change from homopolar to heteropolar bonds triggered by external energy input like photo-, ion-, or γ-ray irradiation, and another is from photo-oxidation^6,7^. For example, PD was found to exist in As_2_S_3_ films^8^, while fast photodarkening and slow photobleaching coexisted in Ge_19_As_21_Se_60_ thin films^9^, where PB was a slower process that started only after PD had saturated. Yang *et*.*al*. showed that, the photosensitivity can be tuned by varying the ratio of Ge/As within the Ge_x_As_45-x_Se_55_ ternary system^10^. Pritam Khan *et al*. first observation of the temperature-dependent photoinduced response of Ge_25_As_10_Se_65_ thin films^11^. Su *et al*. investigated the photosensitivity of Ge_x_As_y_Se_1-x-y_ system, and found that optically stable composition can only exist in the glasses with a mean coordination number of around 2.45 in Ge_x_As_y_Se_1-x-y_^12^. However, these glasses containing toxic arsenic might be burnt or evaporated in the optical experiments when laser power is beyond the damage threshold, this could be harmful to the personal health. Therefore it is highly desirable to investigate photo-induced effects in the glasses that are environmentally friendly.

In Ge_x_Se_100-x_ glasses without containing any arsenic, Kumar *et al*. reported a crossover from PD to PB when the composition of the chalcogenide glassy thin film changes from Ge-deficient to rich^13^. Mishchenko *et al*. observed dynamic variations of the photo-induced effects in amorphous Ge_x_Se_100-x_ films^14^, where a crossover from a transient PD to the mixture of transient PD and metastable PB was found at x = 30 at.%. A.R Barik *et al*. first time showed that ultrafast light illumination can induce an unusually broad transient optical absorption in the sub-bandgap region of chalcogenide GeSe_2_ thin films, which can be interpret as being a manifestation of creation and annihilation of photo induced defects^15^. However, in the present paper, we reported that, with continuous irradiation, PD appears almost instantaneously upon light irradiation, and saturates faster in a shorter time scale, and finally PB becomes dominant at a long time scale. Such an unexpected PB behavior in Ge-deficient Ge_16.8_Se_83.2_ films was subsequently assigned to the structural transformation from ES-Ge(Se_1/2_)_4_ tetrahedral convert to CS-Ge(Se_1/2_)_4_ tetrahedral units upon irradiation and homopolar Ge-Ge and Se-Se bonds to heteropolar Ge-Se bonds.

## Results and Discussions

**Figure 1:**
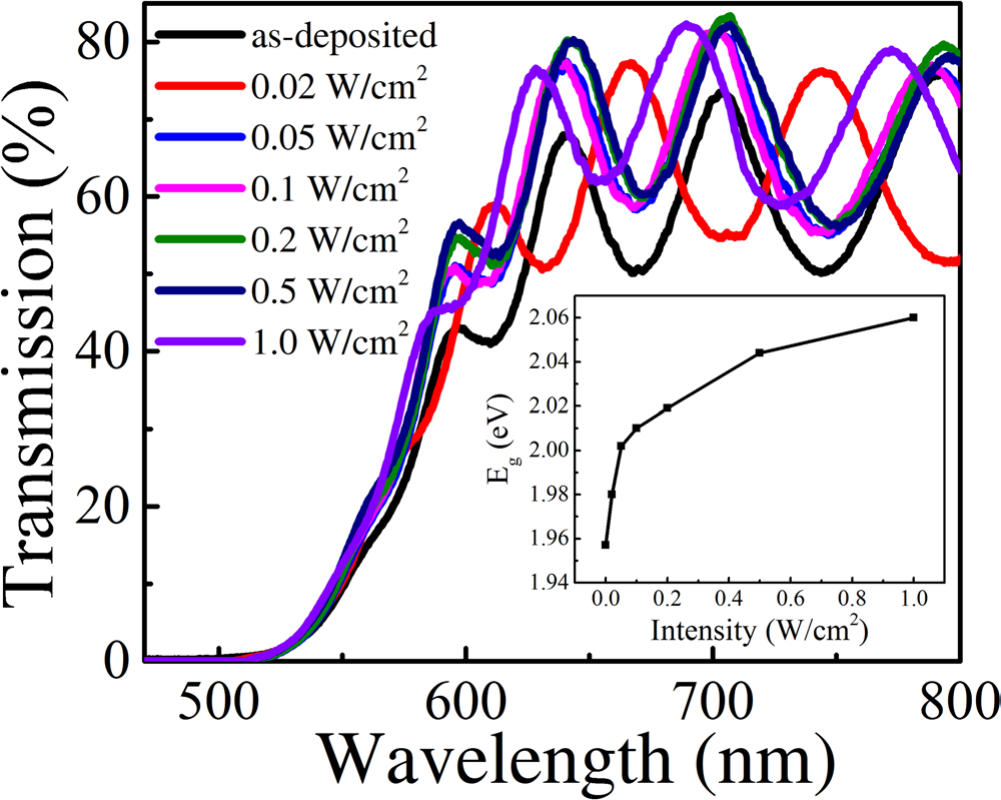
Transmission spectra of as-deposited Ge_16.8_Se_83.2_ film and the film irradiated by a laser with an power density of 0.02, 0.05, 0.1, 0.2, 0.5 and 1 W/cm^2^, respectively. The inset is the bandgap of the films.

The X-ray Diffraction (XRD) patterns indicated that the as-deposited films are amorphous. Smooth surface of the films without any observable particles was also confirm by scanning electron microscope (SEM). The composition of the film was measured to be Ge_16.8_Se_83.2_ by energy dispersive spectroscopy (EDS), and a homogenous distribution of the chemical composition across all the film was confirmed. We examined the effect of the pump beam irradiation on the transmission spectra of the probe beam through the films. We chose the wavelength of the pump beam at λ = 655 nm because it is close to the optical bandgap of the as-deposited and irradiated films. The probe beam was a white light with wavelengths from 400 to 1000 nm, and its power is too low to measure. Therefore, the interference between the irradiation and probe beams can be neglected. Figure 1 showed the transmission spectra of the as-deposited and irradiated films with different laser power density for 12000 s. Generally, as irradiation power density of irradiation increased, absorption edge shifts to a shorter wavelength. The inset of Fig. 1 is the evolution of the optical bandgap as a function of laser power density, where the optical bandgap was calculated using the Tauc’s method. The bandgap was found to increase with increasing power density and the overall PB effect in the film was evident from the inset of Fig. 1.

**Figure 2:**
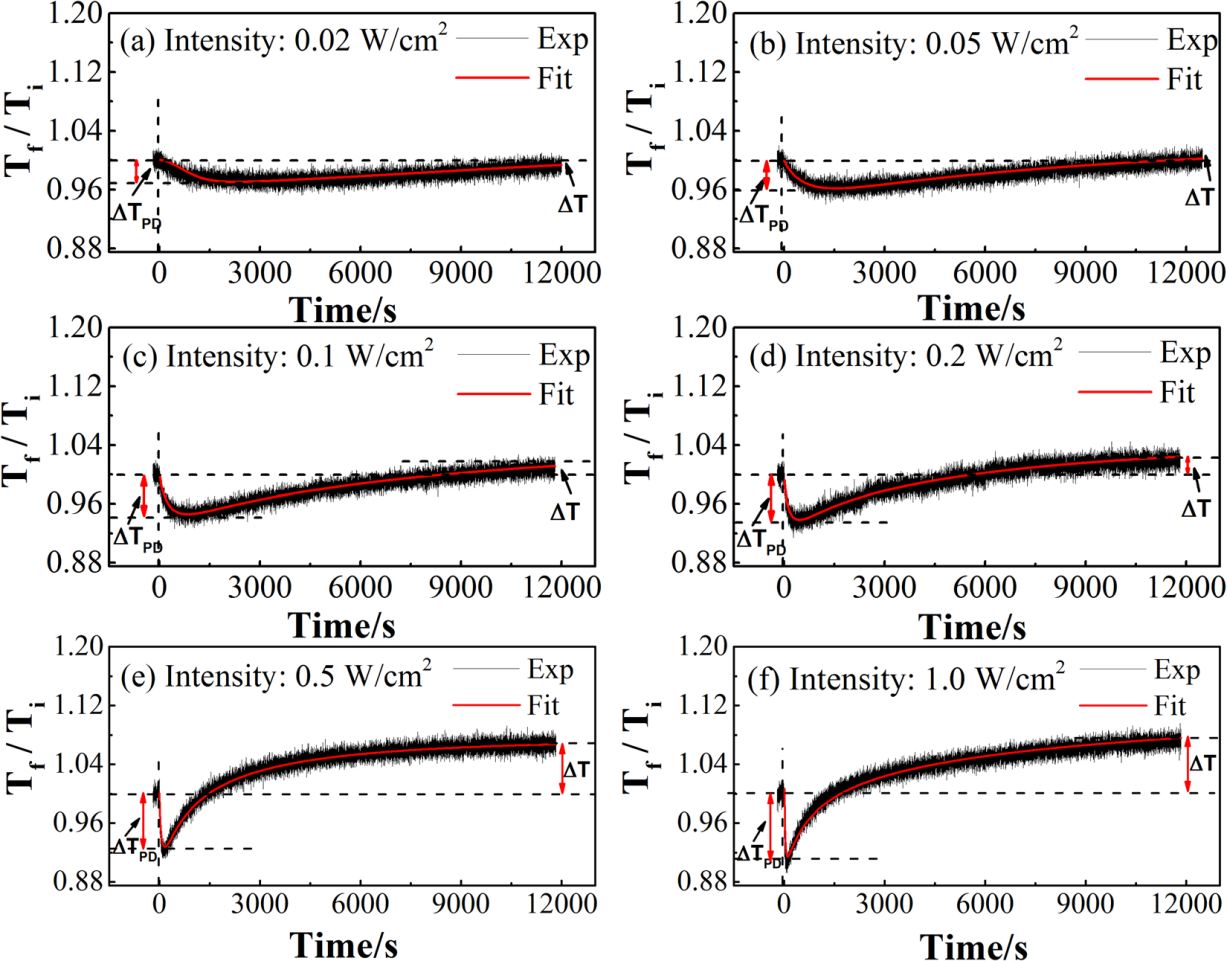
Time evolution of *T*_*f*_*/T*_*i*_ of the films irradiated with a power density of (**a**) 0.02, (**b**) 0.05, (**c**) 0.1, (**d**) 0.2, (**e**) 0.5 and (**f**) 1 W/cm^2^.

In order to further understand the kinetics of photo-induced effects, we evaluated the change in the transmittance of the film with increasing irradiation time. First, we recorded the transmission spectrum of the as-deposited film in the normal conditions and the transmission is defined as *T*_*i*_. Then, we turned on the pump beam and continuously recorded the transmission spectra as a function of time, where the transmission was expressed as *T*_*f*_. In all irradiation-probe experiments, we measured photo-induced response in the different positions of the same film, and observed similar results. Figure 2 shows the time evolution of the transmission ratio *T*_*f*_*/T*_*i*_ of the films irradiated with different power density at a single-probe wavelength of 560 nm. We recorded *T*_*f*_*/T*_*i*_ at this wavelength following ref.^16^ because the transmission at 560 nm was 20% of the value in the dark and as-prepared condition.

Generally, all the spectra exhibit similar trend. In the first 200 s in Fig. 2, we only used the probe beam and found that, *T*_*f*_*/T*_*i*_ is constant. Then we switched on the irradiation beam, it was found that *T*_*f*_*/T*_*i*_ decreases to a minimum value and then increases with increasing irradiation time, corresponding to PD and PB process, respectively. Therefore, the whole kinetic process can be considered as a linear sum of the contributions from both PD and PB. To model the reaction kinetics of opposite photo-induced effects (PD and PB) at six different irradiation power density, we fit the whole process using a combination of the stretched exponential functions that describe PD and PB, respectively^16^:

1 $${\rm{\Delta }}T=A[\exp \{-{({t/\tau }_{d})}^{{\beta }_{d}}\}]+{\rm{\Delta }}{T}_{Sd}+{\rm{\Delta }}{T}_{Sb}[1-\exp \{-{({t/\tau }_{b})}^{{\beta }_{b}}\}]$$

where the subscript *d* and *b* correspond to PD and PB, respectively. *∆T*_*s*_, *τ*, *β*, *t* and *A* are the metastable part, the time constant, the dimensionless parameter, the irradiation time, and a temperature dependent quantity that is equal to the maximum transient changes, respectively. The value, which was determined by the fitting stretched exponential, between the initial process of *T*_*f*_*/T*_*i*_ and the maximum PD was defined as ∆T_PD_, while that the value between the initial and final process of *T*_*f*_*/T*_*i*_ was defined as ∆T.

The fitting parameters based on Eq. (1) are listed in Table 1. From Fig. 2 and Table 1, we observed that, the overall trend of PD and PB remain roughly the same, but both PD and PB processes become dramatically faster with increasing irradiation power density. For example, *τ*_*d*_ increases from 6 to 1000 s and *τ*_*b*_ increases from 32 to 12000 s when the irradiation power density is raised from 0.02 to 1 W/cm^2^, respectively. This clearly indicates that the higher irradiation power density accelerates the kinetics of the PD and PB processes in the films. In any cases, the kinetics of PD are one order faster than that of PB. For example, *τ*_*d*_ is 150 s, while *τ*_*b*_ is 4800 s at irradiation power density of 0.2 W/cm^2^ from Table 1. This verifies that, PD is an instantaneous process with a very short reaction time while PB is a relatively slow process with a longer reaction times.

**Table 1:** Fitting parameters obtained from Eq. (1) with different irradiation power density. The subscript *b* and *d* refer to bleaching and darkening, respectively.

Power density (W/cm^2^)	${{\boldsymbol{\tau }}}_{{\boldsymbol{d}}}$	${{\boldsymbol{\beta }}}_{{\boldsymbol{d}}}$	${\boldsymbol{\Delta }}{{\boldsymbol{T}}}_{{\boldsymbol{S}}{\boldsymbol{d}}}$	${{\boldsymbol{\tau }}}_{{\boldsymbol{b}}}$	${{\boldsymbol{\beta }}}_{{\boldsymbol{b}}}$	${\boldsymbol{\Delta }}{{\boldsymbol{T}}}_{{\boldsymbol{S}}{\boldsymbol{b}}}$
0.02	1200	0.97	0.9545	11000	0.93	0.053
0.05	600	0.95	0.942	10000	0.9	0.085
0.1	280	0.9	0.925	8000	0.85	0.115
0.2	150	0.85	0.907	4800	0.7	0.138
0.5	60	0.7	0.815	950	0.5	0.26
1	36	0.45	0.56	280	0.32	0.53

**Figure 3:**
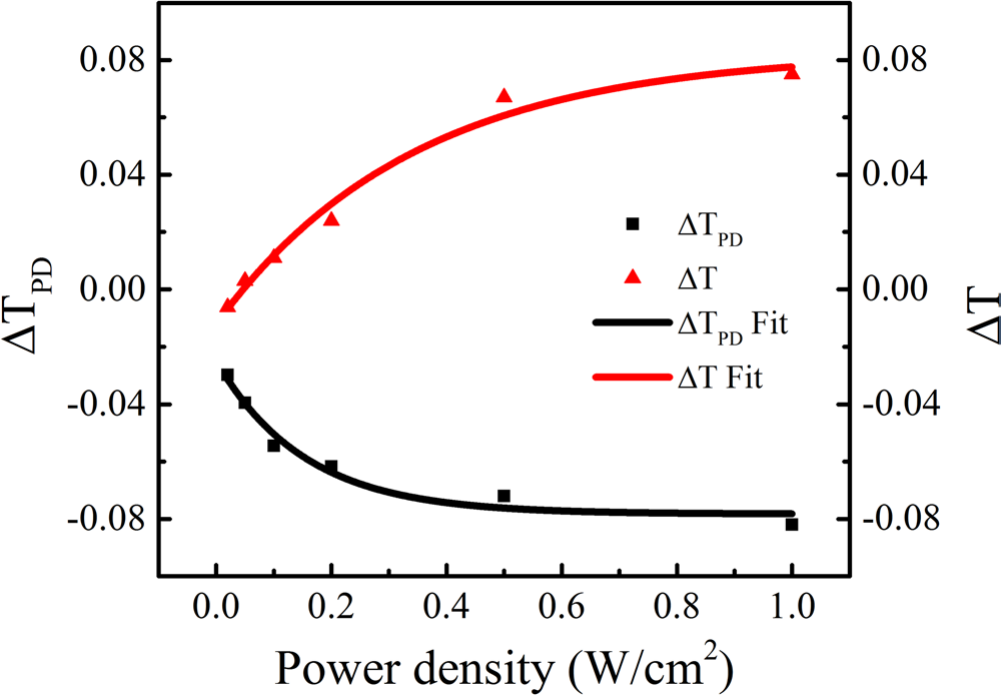
The last amplitude of PD and PB as a function of the irradiation power density.

To quantify the difference between PD and PB, we plotted *ΔT* and *ΔT*_*PD*_ as a function of the irradiation power density in Fig. 3. It was found that, ΔT_PD_ simultaneously appears even at a low irradiation power density of 0.02 W/cm^2^, and it increases to 0.08 at a maximum irradiation power of 1.0 W/cm^2^.

**Figure 4:**
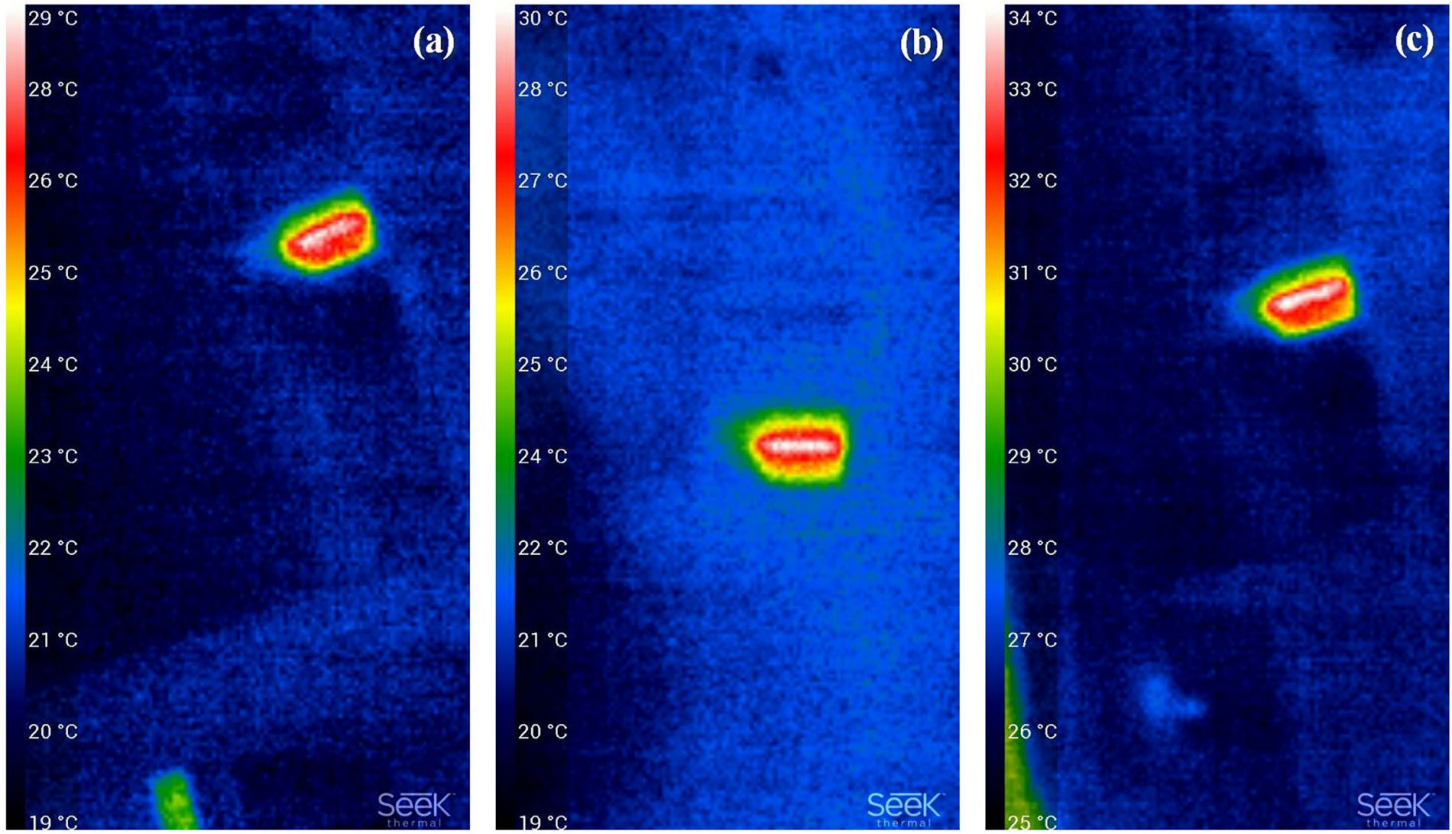
Thermal images of the Ge_16.8_Se_83.2_ film with a pump laser power density of 1 W/cm^2^, (**a**) at the beginning, (**b**) in the middle, and (**c**) at the end of the experiment.

Previous results have showed that, light-induced response in Ge_25_As_10_Se_65_ films is temperature dependent^11^. Therefore, in order to exclude any possibility whether the observed results were induced by the change of the temperature, we further measured the temperature raised by laser irradiation. Figure 4 (a),(b) and (c) are the respective infrared thermal camera images for the films at the beginning, the middle and the end of the experiment. It is evident that, the temperature is around 33–34 °C for the film with 1.0 W/cm^2^ irradiation for 12000 s. This confirms that such a smaller exciting laser power density and large beam spot on the film would result in a relatively insignificant temperature increase during the irradiation. Therefore, we conclude that the observed changes are photo- rather than temperature- induced effect.

Regarding the origin of the PB, either photo-induced oxidation effect or photo-induced intrinsic structural change were suggested^17^. However, photo-oxidation occurs at a much low speed in Ge-deficient chalcogenide thin film^7,13^. We have experimentally demonstrated that the temperature on the film after long time irradiation is still low, and such a temperature is not high enough to accelerate the oxidation process on the surface. Consequently, photo-oxidation is not a major factor to induce PB in the experiments. Therefore, photo-induced intrinsic structural rearrangements might play an important role.

**Figure 5:**
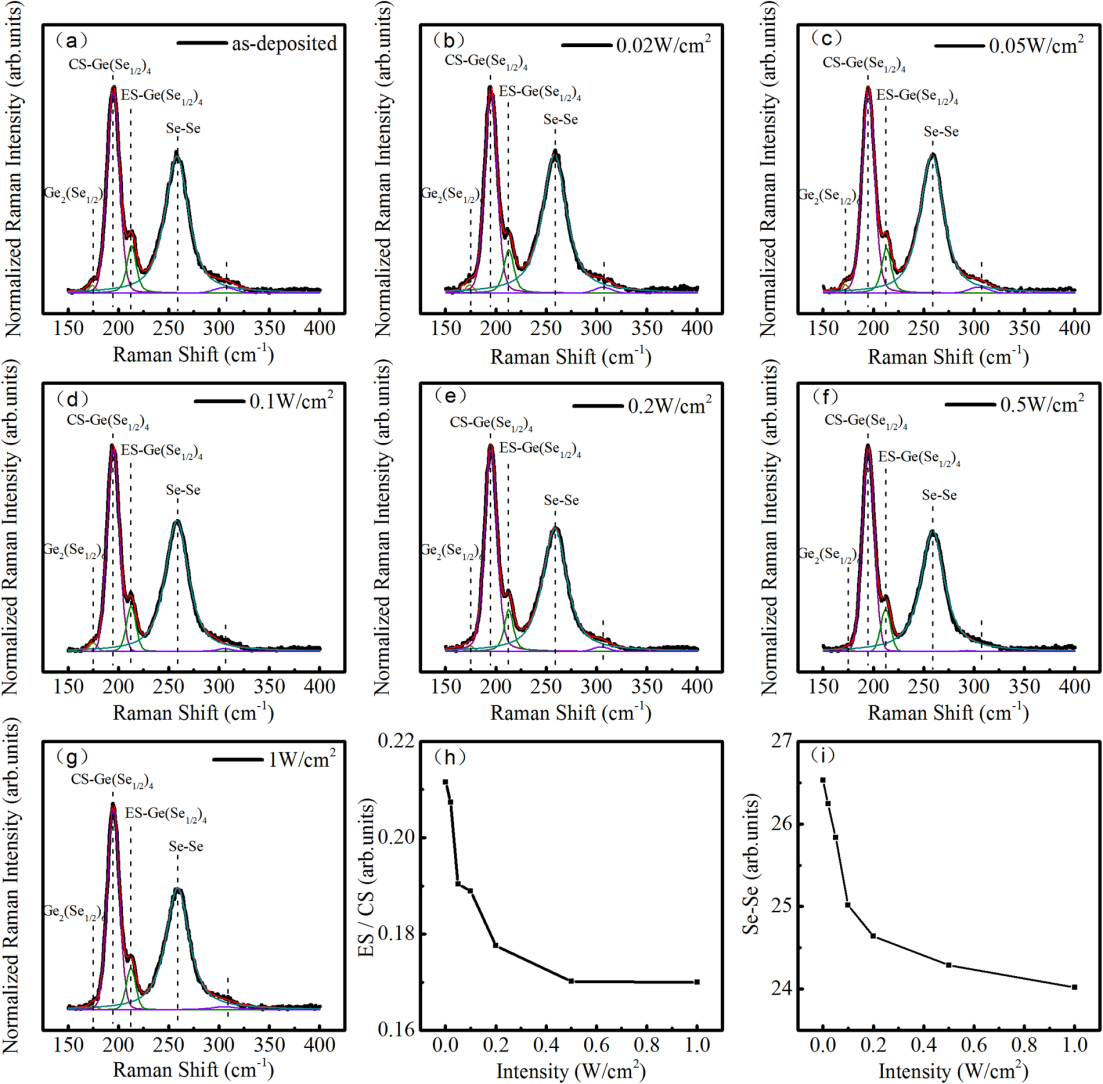
Raman spectra and their decomposition of the (**a**) as-deposited film and the films with a irradiation power density of (**b**) 0.02, (**c**) 0.05, (**d**) 0.1, (**e**) 0.2, (**f**) 0.5 and (**g**) 1 W/cm^2^, respectively. (**h**) Raman integrated area ratio of ES/CS modes. (**i**) Raman integrated area of Se-Se bond.

To examine any possible structural change before and after irradiation, we recorded Raman spectra of the as-deposited and irradiated films, and the results were shown in Fig. 5. The Raman profiles were almost identical for all the films. The peak at 175 cm^−1^ was assigned to the Ge_2_(Se_1/2_)_6_ ethane-like units (ETH bonds), and two main peaks at 197 cm^−1^ and 213 cm^−1^ was ascribed to the Ge(Se_1/2_)_4_ corner-sharing (CS) and edge-sharing (ES) tetrahedral units, respectively. The broad band near 257 cm^−1^ was assigned to the stretching vibrations of Se_n_ chains (Se-Se mode)^18,19^. Figure 5(h) is the ratio of ES-Ge(Se_1/2_)_4_ tetrahedral units and CS-Ge(Se_1/2_)_4_ tetrahedral units. It can be seen that, the ratio decreases with increasing irradiation power density. We therefore conclude that some ES-Ge(Se_1/2_)_4_ tetrahedral convert to CS-Ge(Se_1/2_)_4_ tetrahedral units upon irradiation. This conversion is due to the glass approaching the supercooled liquid equilibrium entropy value through the change of glass structure^20^. It is well known that corner-sharing (CS) tetrahedral structure have lower free energy than the ethane-like unites and edge-sharing (ES) tetrahedral structure in the glass network from the crystal chemistry^19^. Irradiation could also cause some hybridization (mixing) of the lone-pairs and bond p-states at a top of the valence band, this could weaken the Se-Se bond^21,22^. Figure 5(i) is the integrated area of 257 cm^−1^ peak. It was found that, the integrated area decreases with increasing irradiation power density, indicating that part of the Se-Se bonds were broken upon irradiation. Consequently, homopolar bonds like Ge-Ge and Se-Se in the as-deposited film are not stable upon irradiation, and the conversion of these homopolar bonds into the heteropolar Ge-Se bonds occurs via the following process,

2 $$Ge-Ge+Se-Se\mathop{\longrightarrow }\limits^{\hslash \nu }2Ge-Se$$

**Figure 6:**
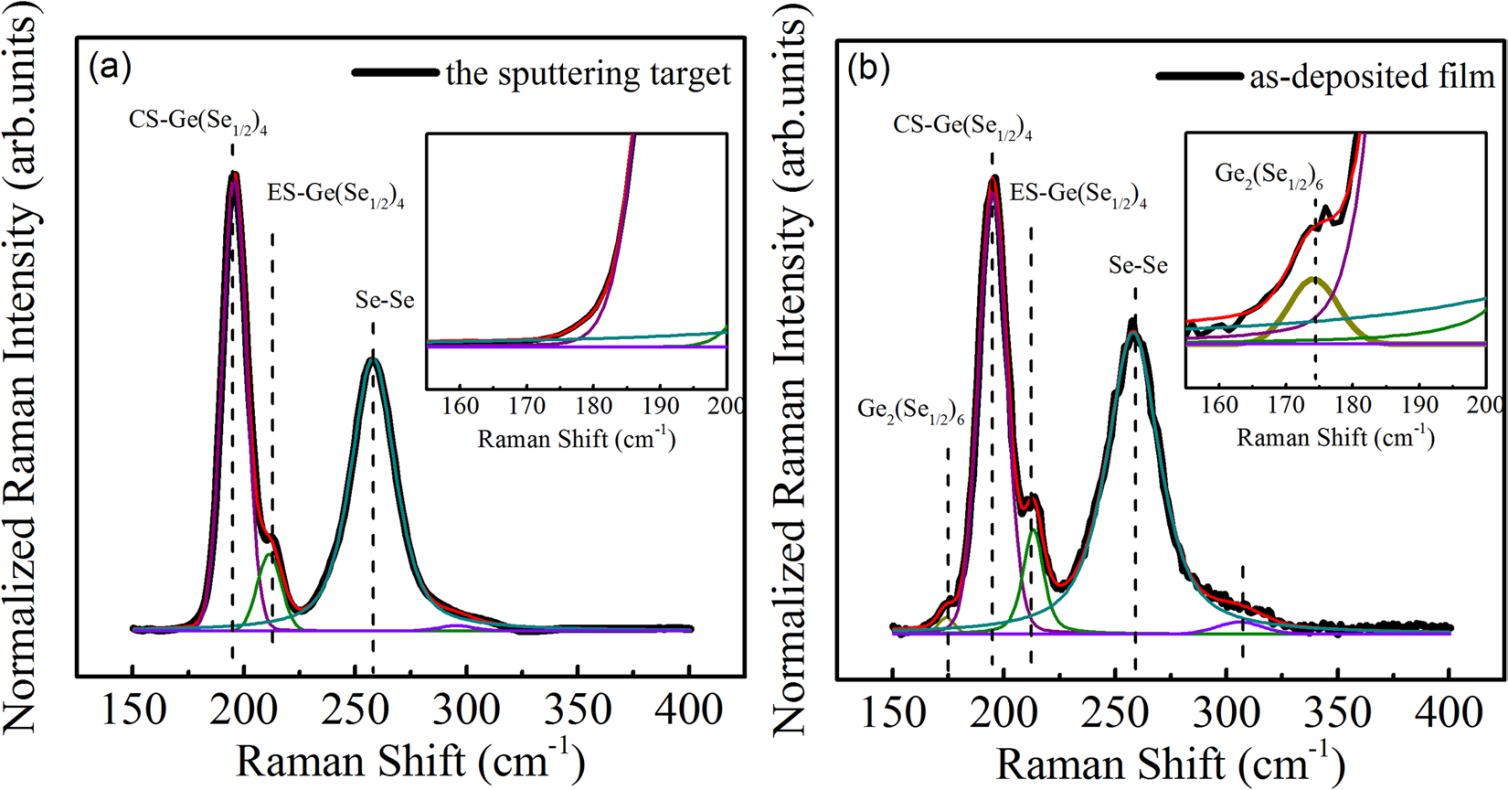
Raman spectra and their decomposition of the target and the as-deposited film.

It is well accepted that, Ge is four- and Se is two-coordinated in GeSe glasses. Therefore, the film used in our experiments is Ge-deficient, located at a so-called floppy phase in Ge-Se glass forming region^23,24^. Such a film usually has a large structural flexibility and thus structural change is much easily induced by photo irradiation. This is one of the reasons why we can probe photo-induced effect even when we used a low irradiation power of 0.02 W/cm^2^. However, unexpected results found in the present paper is that, total PB effect exist in the films with a high irradiation more than 0.1 W/cm^2^, which is in sharp contradictory with the previous observation that only PD exist in the Ge-deficient film. We have carefully examined the chemical composition of the films and recorded the temperature induced by laser irradiation, and thus excluded any possible artifacts. Moreover, the results clearly showed that the photo-induced effect can be further enhanced with increasing irradiation power density. Previously, Mishchenko *et al*. ascribed the increase in transient PD amplitude with increasing Ge content to the suppression of the concentration of Se lone pair states, and the final increase in the metastable PB amplitude to the increase in the number of the ETH bonds in Ge_36_Se_64_ glasses^14^. In the present experiments, the amplitude of the transient PD increases with increasing irradiation power density as shown in Fig. 3, since more homopolar Se-Se bonds would be broken and heteropolar Ge-Se bonds would be formed. However, it is plausible to ascribe the increase in PB amplitude in Fig. 3 to the ETH bonds. To examine the different of the structural, we compared Raman spectra of the target and the as-deposited film, and the results were shown in Fig. 6. We noted the fact that, the Ge_16.8_Se_83.2_ films prepared by the magnetron sputtering method contain more ETH bonds compared with the target, although the nominal composition of the film is more Ge-deficient. Generally, such ETH bonds cannot be expected in Ge-deficient bulk glasses^20,25^. However, it is possible to find Ge-Ge bonds in Ge-deficient films since the films are created in the nonequilibrium conditions where more defective bonds can be expected. We cannot find any significant change of the ETH contents during the irradiation as shown in Fig. 5. Nevertheless, compared with thermal evaporation where the glasses were decomposed into molecules or clusters, magnetron sputtering usually decompose the glasses into various ions, then recondense on the surface, and this inevitably creates more defective dangling bonds. We believe these defects are major reasons why we observe such an unexpected PB behaviour in Ge-deficient film. The correlation between these defects and the photoinduced effects, and whether the observed results are sensitive to the films prepared by different methods, will be further explored in the near future.

## Conclusion

In summary, we have demonstrated that coexistence of fast PD and slow PB in Ge-deficient chalcogenide glassy thin films, and observed that the degrees of PD and PB processes are directly related to the irradiation power density. These photoinduced effects saturate at a larger value which is well above the initial value when irradiated by a higher irradiation power density. Raman spectra provide direct evidence that such photoinduced effects are related with the change of the glass structure. The present results are in sharp contrast with the previous one, where the PB process can only be observed in Ge_x_Se_100-x_ film with x > 30. This opens up a new approach to tune the photoinduced effects in chalcogenide glasses via tuning chemical compositions or irradiation power.

## Method

The sputtering targets was prepared by the melt-quench method using 5 N Ge and Se powders. The films were grown on quartz and Si substrates by the radio-frequency magnetron sputtering method. The chamber was evacuated to 5.0 × 10^–4^ Pa, and then flowing Ar gas was introduced to the chamber. The pressure during the deposition was set to 0.3 Pa. The compositions of the films were determined by the energy dispersive spectroscopy (EDS). The film thickness was *in-situ* controlled by a thickness monitor equipped in the chamber and further *ex-situ* measured by Veeco Dektak 150 surface profiler, being about 1 μm, which was approximately the penetration depth of band-gap light for the film. The amorphous nature of the films was confirmed by X-ray Diffraction (XRD) patterns.

The experimental setup for photo-induced changes was the same as that used in ref.^26^. We have chosen the wavelength of the pump beam as λ = 655 nm (from a diode pumped solid state laser, DPSSL) as the pump beam with an elliptic spot where the long and short axis is 4 and 2mm, respectively. The pump power density varied from 0.02, 0.05, 0.1, 0.2, 0.5 to 1 W/cm^2^. The probe beam was a low power density white light with a wavelength from 400 to 1000 nm. The probe white light beam was overlapped with the pumping laser spot on the surface of the film but the beam size of the former one was smaller than that of the latter one. During the irradiation, the transmission of the sample was recorded using an Ocean Optics high resolution composite grating spectrometer (HR 2000 CG-UV-NIR). All irradiation-probe experiments were performed at the as-deposited films.
