# Publisher Correction: Observation of photobleaching in Ge-deficient Ge_16.8_Se_83.2_ chalcogenide thin film with prolonged irradiation

**DOI:** 10.1038/s41598-018-20339-8

**Published:** 2018-01-23

**Authors:** Sen Zhang, Yimin Chen, Rongping Wang, Xiang Shen, Shixun Dai

**Affiliations:** 1 Laboratory of Infrared Materials and Devices, Ningbo University, Ningbo, Zhejiang 315211 China; 2 Key Laboratory of Photoelectric Detection Materials and Devices of Zhejiang Province, Ningbo, 315211 China; 3 University of Chinese Academy of Sciences, Beijing, 100049 China; 4 Laser Physics Centre, The Australian National University, Acton, Canberra, ACT 2605 Australia

Correction to: *Scientific Reports* 10.1038/s41598-017-14796-w, published online 03 November 2017

The original HTML version of this Article contained a typographical error in the publication date ‘03 November 2017’ which was incorrectly given as ‘06 November 2017’. This has now been corrected in the HTML version of the Article.

